# Supplementary material for: Inconsistency among evaluation metrics in link prediction
Source: PNAS Nexus. 2024 Nov 6;3(11):pgae498. doi: 10.1093/pnasnexus/pgae498 (PMC11574622; doi:10.1093/pnasnexus/pgae498)
Supplement: pgae498_Supplementary_Data [file pgae498_supplementary_data.pdf]

Inconsistency among evaluation metrics in link prediction

Yilin Bi, Xinshan Jiao, Yan-Li Lee, Tao Zhou

1 Sensitivity Analysis

We first test the impacts of the ratio of the training set to the probe set. In addition to the commonly used ratio, say $|E^T|$: $|E^P|= 9 : 1$, we consider other ratios like 8:2, 7:3 and 6:4, which are also usually used in binary classification. As shown in Figure S1, the change in ratio has tiny effects on the correlations between metrics, suggesting the robustness of observations in the main text. We next check whether our results are sensitive to the choice of correlation measures by comparing the Kendall rank correlation coefficient with another well-known coefficient, say the Spearman rank correlation coefficient. As shown in Figure S2, they show the same trend, indicating that our results are robust to the correlation measures.

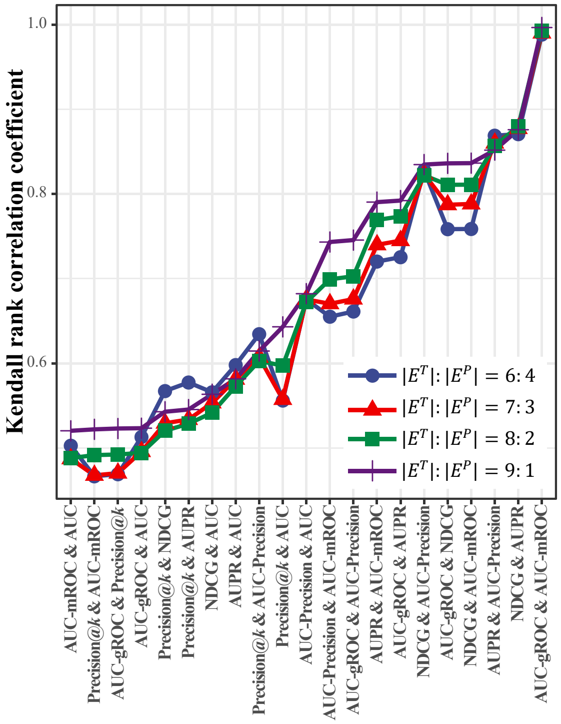

Figure S1: The average pairwise correlations over 300 randomly selected real networks for different splitting ratios of $|E^T|$ to $|E^P|$. The blue, red, green, and purple lines represent the results for $|E^T|$: $|E^P|= 6 : 4$, $|E^T|$: $|E^P|= 7 : 3$, $|E^T|$: $|E^P|= 8 : 2$, and $|E^T|$: $|E^P|= 9 : 1$, respectively.

2 Deviation Analysis

In order to compare the variability among multiple experimental outcomes, we computed the standard deviation (i.e. error bars) of the case with Q = 300, as presented in Table S1. In this table, there error bars are very small compared to the mean correlations and do not affect the conclusions of this work.

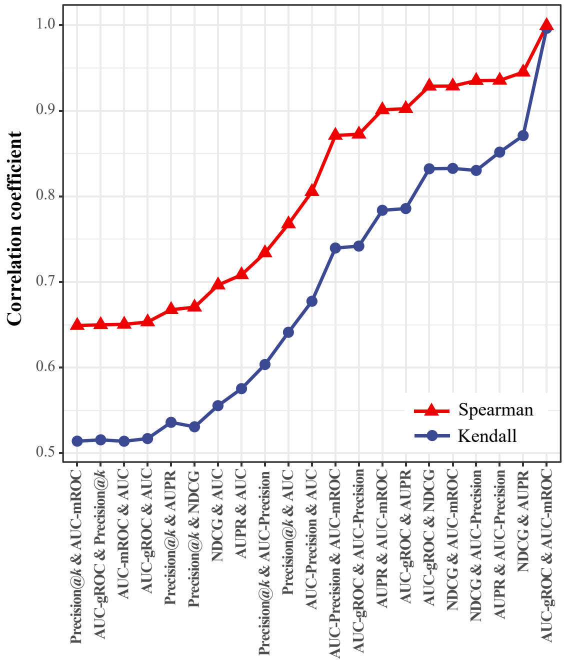

Figure S2: The average pairwise correlations over 300 randomly selected real networks, obtained by different correlation measures. The red and blue lines represent the results obtained by the Kendall rank correlation coefficient and the Spearman rank correlation coefficient, respectively.

3 Principal Component Analysis

Each metric is initially represented by a 26-dimensional vector that records the average ranks of the 26 algorithms, then we apply Principal Component Analysis (PCA) on those vectors to visually show the closeness of any two evaluation metrics. As shown in Figure S3, evaluation metrics primarily cluster into three groups. AUPR, AUC-Precision, NDCG, AUC-mROC and AUC-gROC form one cluster, indicating a close relationship among these five metrics, all the threshold-dependent metrics are identical to each other, and AUC stands alone. Overall speaking, two evaluation metrics with higher correlation has shorter distance in the PCA plot. These results are essentially consistent with our findings in the main text (see Figure 4 in the main text).

4 Negative Sample

We consider the balanced learning case where the number of negative samples is set to be $|E^P|$, the same to the number of positive samples. As shown in Figure S4, except for the correlation between AUC and AUC-gROC that is close to 1, the correlations for the other 20 pairs are significantly less than 1. This once again indicates that most pairwise metrics remain inconsistent with each other.

5 Alternative Method

Different from the method in Figure 1 of the main text, there is an alternative way to calculate the correlation between metrics based on a large number of real networks. The key point of this method is to first average the ranks of different algorithms over selected networks, and then to calculate the Kendall rank correlation coefficient of the mean ranks. The different part of this method from the

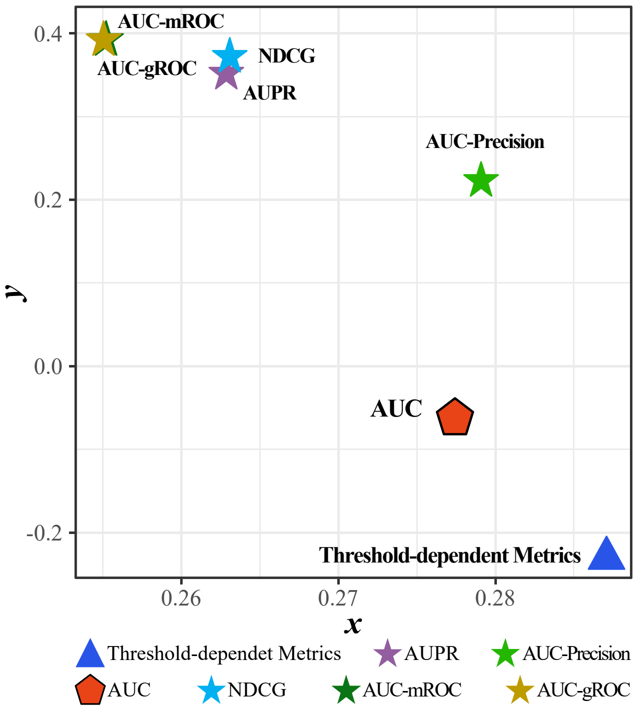

Figure S3: PCA of the rankings of the 26 algorithms associated with these evaluation metrics. The x-axis represents the scores for the first principal component, and the y-axis represents the scores for the second principal component. The positions for threshold-dependent metrics (i.e. Precision@k, Recall@k, MCC@k, Specificity@k, Youden@k, Accuracy@k, F1@k, with $k = 0.1 \cdot |U - E^T|$) are identical, represented by a blue triangle.

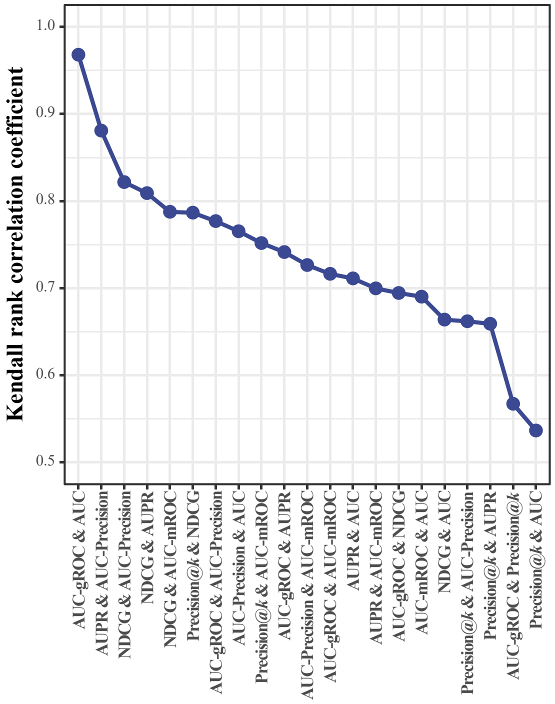

Figure S4: The Kendall rank correlation coefficients for all metric pairs, averaged over 300 selected networks. The number of negative samples is set to $|E^P|$, and the threshold is set to $|E^P|$

Table S1: The mean correlation and corresponding standard deviation for each metric pair when $Q = 300$.

**Metric Pair**	**Mean Correlation**	**Standard Deviation**
AUC-gROC and AUC-mROC	0.996578	0.000405
NDCG and AUPR	0.875870	0.003853
AUPR and AUC-Precision	0.851775	0.002680
NDCG and AUC-mROC	0.836409	0.002043
AUC-gROC and NDCG	0.836183	0.002168
NDCG and AUC-Precision	0.834747	0.001958
AUC-gROC and AUPR	0.792213	0.003451
AUPR and AUC-mROC	0.790380	0.003596
AUC-gROC and AUC-Precision	0.745558	0.001798
AUC-Precision and AUC-mROC	0.743283	0.001999
AUC-Precision and AUC	0.682267	0.003807
Precision and AUC	0.643151	0.003747
Precision and AUC-Precision	0.614595	0.004330
AUPR and AUC	0.581726	0.005182
NDCG and AUC	0.563456	0.004215
Precision and AUPR	0.545441	0.003841
Precision and NDCG	0.542834	0.003919
AUC-gROC and AUC	0.523385	0.003384
AUC-gROC and Precision	0.523030	0.004053
Precision and AUC-mROC	0.522024	0.003928
AUC-mROC and AUC	0.520339	0.003301

method applied in the main text is shown in Figure S5. Figure S6 compares the results of the first and second methods for the toy model introduced in the main text. For the first method (see Figure S6A), when Q is large enough, the correlation coefficient between X and Y stabilizes at about 0.49. However, for the second method (see Figure S6B), the correlation coefficient rapidly increases to 1 as Q increases. Figure S7 reports the results by using the second method for real networks, where the other settings are completely the same to those of Figure 4 in the main text.

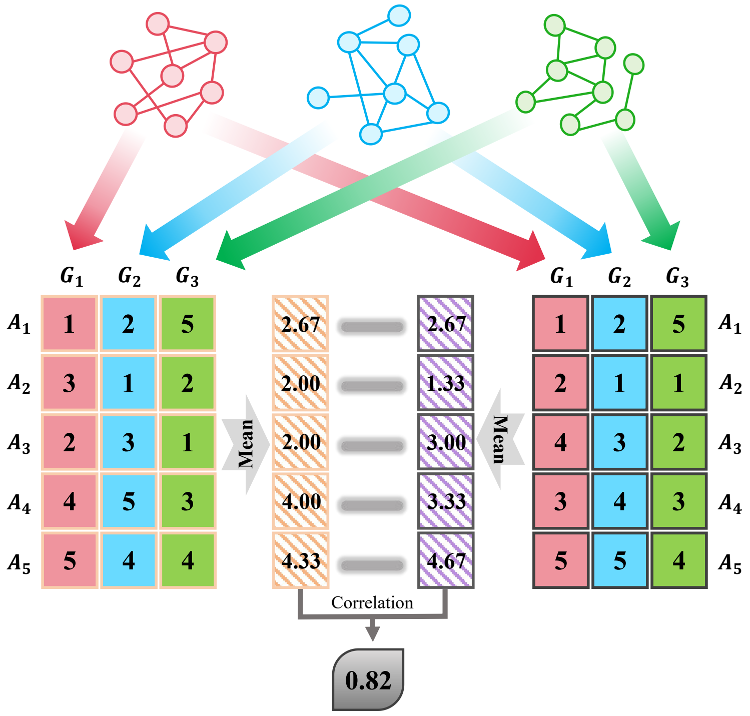

Figure S5: Schematic flowchart of an alternative averaging method to measure the correlation between any two evaluation metrics M_1 and M_2 for five algorithms ($P = 5$). After obtaining the rankings of algorithms for the Q selected networks (here we show an example for $Q = 3$), we first calculate the mean ranks and then measure the correlation between two vectors of mean ranks.

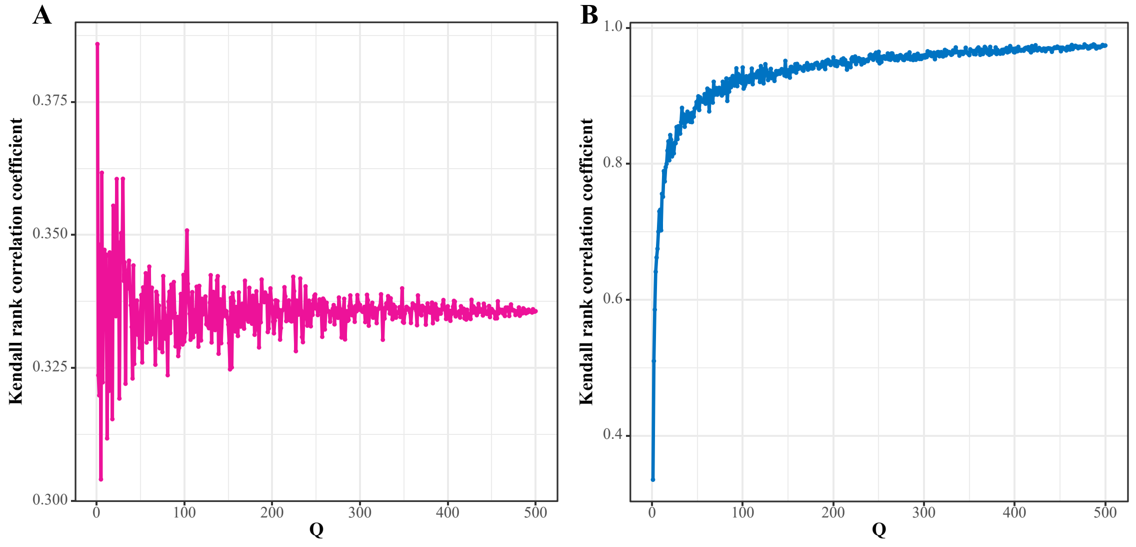

Figure S6: The Kendall rank correlation coefficients between X and Y as the increasing of Q for the toy model using (A) the first method and (B) the second method.

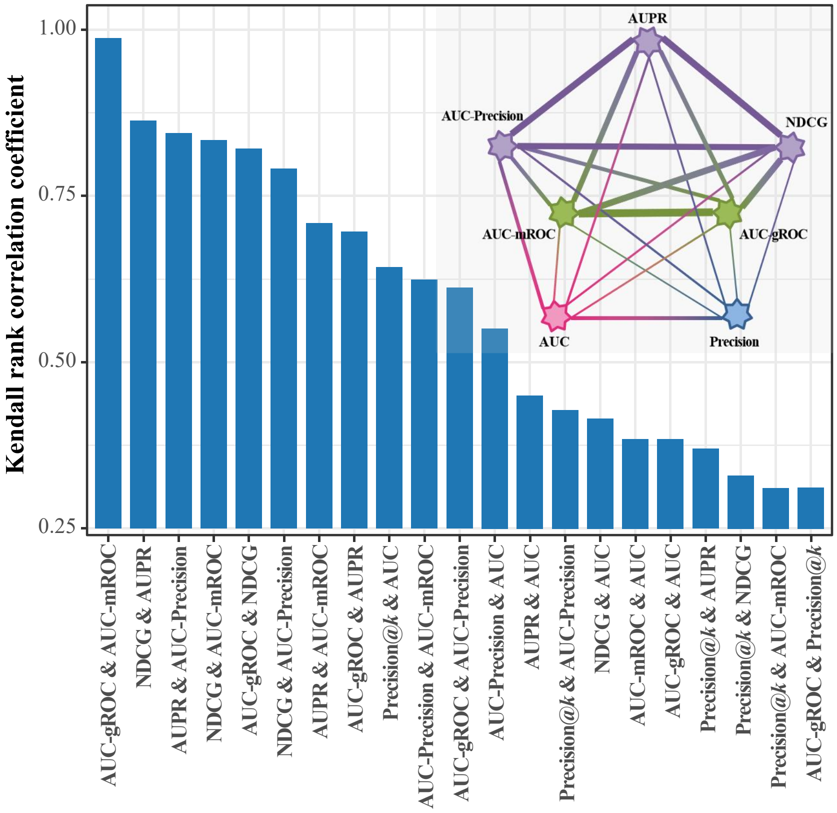

Figure S7: The Kendall rank correlation coefficients for all metric pairs, obtained by the method presented in Figure S5, which are averaged over 10 independent runs and 300 selected networks in each run. For Precision, the threshold is set as $k = 0.1 \cdot |U - E^T|$. The top-right corner shows the corresponding correlation graph, with the thickness of each link representing the strength of correlation.
